# 336. COVID-19 and *Pneumocystis jiroveci* Pneumonia

**DOI:** 10.1093/ofid/ofab466.537

**Published:** 2021-12-04

**Authors:** Christopher Saling, Sabirah N Kasule, Holenarasipur R Vikram

**Affiliations:** 1 Mayo Clinic in Arizona, PHOENIX, Arizona; 2 Mayo Clinic hospital, Phoenix, Arizona, Phoenix, AZ

## Abstract

**Background:**

More accounts of opportunistic infection in COVID-19 patients are emerging. At our institution, we identified 2 COVID-19 patients with *Pneumocystis jiroveci* pneumonia (PJP) opportunistic infection. This prompted a review of the literature to identify trends in patient characteristics, risk factors, and outcomes in this population.

**Methods:**

A literature review was conducted using PubMed that identified 13 other patients with both COVID-19 and PJP infection. Age, gender, human immunodeficiency virus (HIV) status, other immunocompromised states, time between COVID-19 and PJP diagnosis, and clinical outcomes were captured for analysis.

**Results:**

Eleven patients were male. The average age was 56 years. All but 2 patients were immunocompromised. At time of PJP diagnosis, seven patients had newly diagnosed HIV and one had known, well-controlled HIV. One patient had rheumatoid arthritis receiving leflunomide, 1 had ulcerative colitis receiving budesonide and sulfasalazine, 2 patients had multiple myeloma whereby both were on lenalidomide, 1 patient was a renal transplant recipient immunosuppressed on tacrolimus, mycophenolate, and methylprednisolone, and 1 patient had chronic lymphocytic leukemia getting fludarabine, cyclophosphamide, and rituximab. Nine patients had positive COVID-19 and PJP tests performed within 7 days of one another. One patient tested positive for PJP 54 days into admission for COVID-19. This patient received high dose steroids and tocilizumab for initial COVID-19 infection. Three patients were re-hospitalized with PJP after a recent admission for COVID-19 pneumonia, with a mean time to readmission of 25 days. One of these 3 patients had no treatment for COVID-19, while 2 received steroids. Five of the total 15 patients (33%) died.

**Conclusion:**

COVID-19 treatments with high dose steroids and tocilizumab can make patients vulnerable for opportunistic infection with PJP. Furthermore, COVID-19 is known to cause lymphopenia which may further increase this risk. A diagnosis of concomitant PJP can be especially challenging due to nearly identical radiographical findings. Serum beta-D glucan and HIV testing can be especially helpful in this situation, and there should be a low threshold for performing bronchoalveolar lavage.

**Disclosures:**

**All Authors**: No reported disclosures

